# Management of Variceal Hemorrhage

**DOI:** 10.4021/gr2009.02.1275

**Published:** 2009-01-20

**Authors:** Yan Li, Chun Qing Zhang

**Affiliations:** a Department of Gastroenterology, Provincial Hospital affiliated to Shandong University, Jinan 250021, China

**Keywords:** Variceal hemorrhage, Endoscopy, Management, Prophylaxis

## Abstract

Variceal hemorrhage is a frequent and lethal complication of portal hypertension. Bleeding occurs in 30%-40% of patients with cirrhosis and varices. The first episode of variceal bleeding is associated with a high mortality as well as a high incidence of re-bleeding. Thus, management of variceal hemorrhage should be categorized into 3 phases: primary prophylaxis (prevention of the first episode of bleeding), emergency treatment (management of acute bleeding), and secondary prophylaxis (prevention of re-bleeding). Modalities involved include pharmacological, endoscopic, surgical, interventional radiological therapy and balloon tamponade. This review summarizes the current choices of management during each phase, and concentrates on the following questions, what can we do to prevent the formation and development of varices; how can we predicate the risk of bleeding; what should we do in case of bleeding; what is the first-line therapy; what should we do when current therapy fails; when should we give up and what is the optimal strategy for secondary prophylaxis.

## I. Introduction

Variceal hemorrhage is a frequent and lethal complication of portal hypertension. Esophageal varices are present in around 50% of cirrhotic patients [1]. Bleeding occurs in 30% - 40% of cirrhotic patients once varices have formed [2]. The first episode of variceal bleeding is associated with mortality between 17% - 57% [1], and approximately two thirds of the survivors who do not receive active treatment might suffer from recurrent episode of hemorrhage [2]. Therefore, management of varices can be categorized into three phases, primary prophylaxis (prevention of the first episode of bleeding); emergency treatment (management of acute bleeding); and secondary prophylaxis (prevention of re-bleeding).

During the last decades, management of variceal hemorrhage has been well developed. In 1939, endoscopic injection sclerotheropy emerged; quinine was used as the sclerosant. In the 1970s, interventional radiology procedures, including transportal obliteration, left gastric artery embolization, and partial splenic artery embolization, were introduced. In 1986, endoscopic variceal ligation was first used by Stiegmana. These developments have remarkably improved survival of variceal bleeding. The choosing of these therapies becomes an attractive question.

## II. Primary Prophylaxis of Variceal Hemorrhage

**Figure 1:**
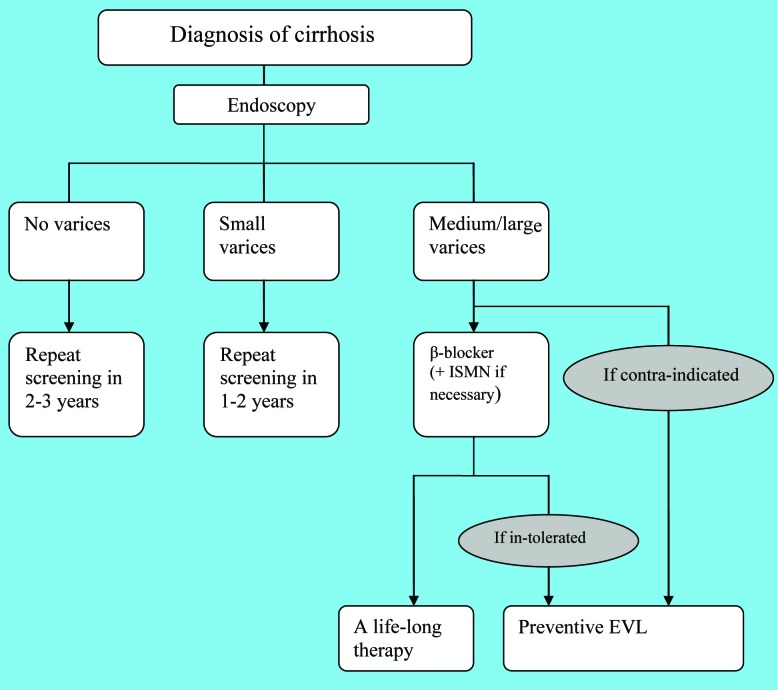
The management of primary prophylaxis

The first episode of variceal bleeding is associated with not only a high mortality but also a high recurrence of bleeding [2]. Hence, prevention of the first episode of hemorrhage is of vital importance. Factors related to the risk of variceal bleeding include portal pressure, endoscopic features of varices and the location of varices [2-4]. Thus, screening the development of varices may help predict the risk of bleeding. Primary prophylaxis of variceal hemorrhage involves reasonable surveillance strategies and appropriate choice of therapeutic modalities. The management of primary prophylaxis is illustrated in Figure 1.

### Surveillance strategies

Varix is a progressive complication of portal hypertension. Thus, surveillance strategies are vitally important since the management of esophageal varices largely depends on its natural history. Hepatic venous pressure gradient (HVPG) is a promising predicative marker of the first episode of bleeding. But its application is limited given its invasive nature. Screening endoscopic is the recommended form for surveillance [5].

#### HVPG

HVPG is a reliable parameter of portal pressure given the positive correlation between variceal pressure and HVPG value [6]. The normal HVPG value is 1 - 5 mmHg. Pressure exceeding the upper threshold defines the presence of portal hypertension, regardless of clinical manifestations. HVPG ≥10 mmHg is defined as clinically significant portal hypertension, predicating the development of varices [7]. HVPG above 12 mmHg is the threshold pressure that leads to variceal rupture [2, 3, 7, 8, 9]. In other words, if HVPG can be lowered to less than 12 mmHg, bleeding does not occur. Overall, the HVPG has predictive value in the development of varices and the risk of bleeding. It is also helpful in assessing the therapeutic efficacy of β-blockers [2, 5, 10]. However, considering the invasive nature, sequential measurements of HVPG are rarely used in practice [4, 5].

#### Endoscopy surveillance

There are several non-invasive markers reported which are related to the risk of bleeding, such as platelet count, diameter of portal vessel, size of spleen, and so on. However, these markers could offer less predictive accuracy. Endoscopy is now the recommended form of screening [5]. Esophagogastroduodenoscopy (EGD), the gold standard in the diagnosis of varices, should be performed once the diagnosis of cirrhosis is established [5, 11]. The morphological features at the initial endoscopy, as well as the natural history of cirrhotic patients, determine the schedule of endoscopy surveillance. For patients with compensated cirrhosis, screening endoscopy should be repeated every 1 - 2 years in individuals with small varices [3-5, 9, 12], while it is reasonable to repeat at 2 - 3 years’ intervals in those without varices [3, 5, 9, 11, 12]. Patients with medium or large varices or red wale signs are generally considered to correlate with high risk of bleeding [13], it is suggested that this group should take non-selective β-blockers for primary prophylaxis [1, 3-5, 11-14]. Endoscopy surveillance can be avoided in these patients [5]. Once there is evidence of decompensate cirrhosis, screening endoscopies should be performed annually in order to monitor the formation and progression of varices [5, 9].

#### Esophageal capsule endoscopy

Esophageal capsule endoscopy (ECE) has opened a new era in variceal examination. First introduced in 2000, ECE is considered as a promising alternative to EGD for patients who are unwilling or unable to undergo EGD [5, 9, 15]. ECE is associated with minimal invasiveness which is responsible for good tolerance [15], and the good agreement with EGD has been certified [9, 15]. However, it is expensive and further studies are required to confirm its application.

### Therapeutic strategies

The non-selective β-blockers, including propranolol and nadolol, are the recommended agents for primary prophylaxis of variceal hemorrhage [8, 16]. It is believed that they can reduce portal pressure by reducing cardiac output as well as splanchnic arterial blood flow [4, 9, 17]. Thus, the selective β-blockers are considered ineffective in prevention of variceal hemorrhage if it doesn’t affect splanchnic circulation [8]. The therapy usually starts with a low dose and then increases to an optimal dose step by step [9, 16]. As discussed above, sequential measurements of HVPG are not widely used in assessing the therapeutic response of β-blockers, therefore, most clinicians empirically adjust the dosage via observing the reduction in heart rate. The optimal dosage is generally considered to be able to reduce the resting heart rate by 25%, or to reduce the resting heart rate to 55 beats per minute [9, 16]. However, the reduction in heart rate does not virtually correlate with the decrease in portal pressure [9, 16].

The contraindications for non-selective β-blockers include asthma, pulmonary edema, chronic obstructive pulmonary disease, congestive heart failure, bradycardia, atrioventricular block, Raynaud’s phenomenon and poorly controlled diabetes mellitus. Nadolol is a preferred agent with fewer adverse effects [8, 16]. Considering these drawbacks, patients with small varices or without varices do not need to take non-selective β-blockers for primary prophylaxis; only patients with medium or large varices take it as the current agent to prevent the first episode of hemorrhage. The nitrates, such as isosorbide mononitrate (ISMN), could further reduce portal pressure [9, 17], however, it is now no longer used in mono-therapy, it is recommended to be used with β-blockers when patients are not adequately sensitive to β-blockers [1, 8, 16]. Moreover, approximately 30% of patients with large varices have contraindication or intolerance to β-blockers [9, 17]. Endoscopic variceal ligation (EVL) is the option of endoscopic treatment when pharmacological therapy is infeasible [1]. Endoscopic injection sclerotherapy (EIS) has no role in primary prophylaxis of variceal hemorrhage, so it is the same with transjugular intrahepatic portosystemic shunting (TIPS) and shunt surgery [1, 8, 9].

## III. Emergency Treatment of Variceal Hemorrhage

**Figure 2:**
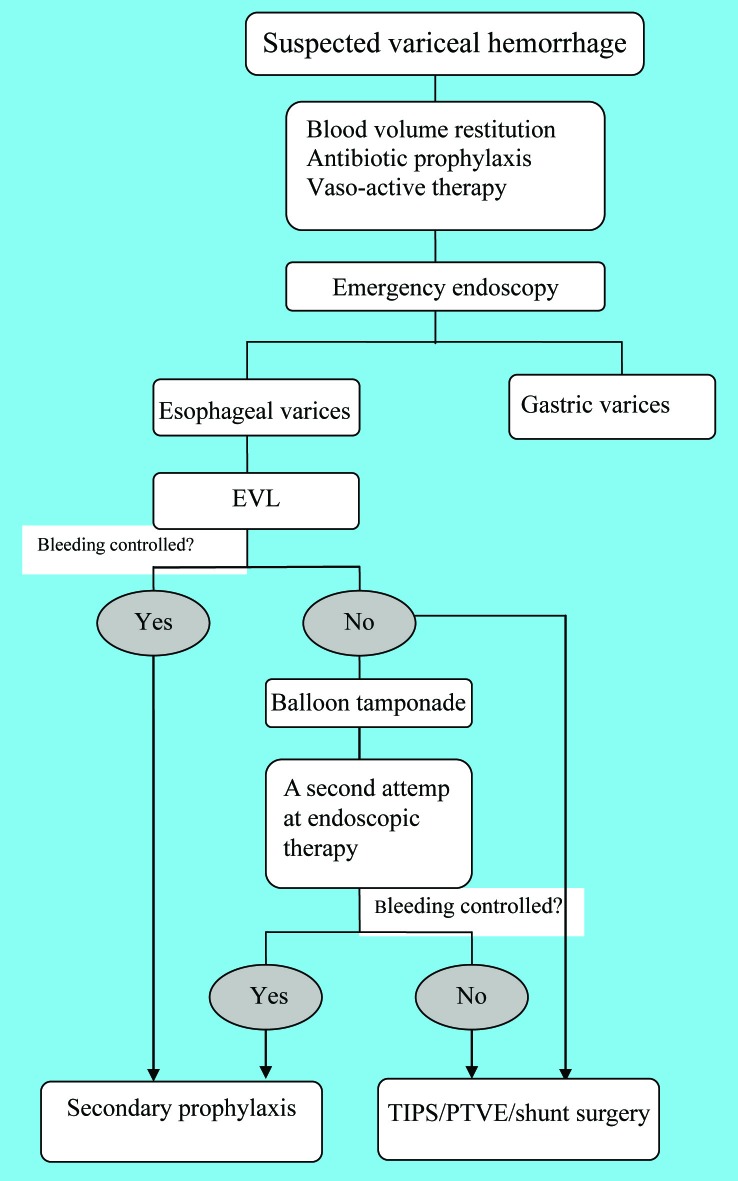
Algorithm for the emergency management

The emergent management of acute variceal bleeding consists of multiple steps, initial resuscitation, emergency endoscopy for diagnosis, hemostasis and prevention of early complications (including bacterial infections and renal failure). The current therapy fails to control acute bleeding or prevent very early re-bleeding in approximately 10% - 20% of the patients [2, 5, 9], in such cases, timely justification of the therapeutic strategy is of vital importance. The emergency management of variceal hemorrhage is illustrated in Figure 2.

### Initial resuscitation

Variceal hemorrhage classically presents as massive upper digestive bleeding with hematemesis or melena. Hemodynamic instability is responsible for many life-threatening complications, such as shock and renal failure. Thus, initial resuscitation is quite crucial.

Hemodynamic restitution should be initiated as soon as possible [2, 18]. After the initial assessment of blood loss, volume replacement should be employed at once. A protected air way is necessary [3, 19]. Endotracheal intubation might be required given that aspiration may take place especially in patients with hepatic encephalopathy or uncontrolled massive bleeding [2, 18, 19]. Peripheral venous access need to be prepared for volume replacement [3]. A central venous line is helpful to monitor central venous pressure which is the guidance of volume resuscitation [3].

Volume replacement should be individualized according to clinical manifestation, age, cardiac function, etc [18]. Gelatin-based colloids, human albumin fractions, fresh frozen plasma and packed red blood cells (PRBC) are widely used for transfusion [9, 18, 20]. Generally, normal saline, dextrans, hydroxyethyl starch and Ringer’s lactate solution are believed to be avoided (Table 1). The transfusion of fresh frozen plasma and platelet should be employed in case of coagulopathy and significant thrombocytopenia (platelet count < 5 × 10^4^ /ml), which are commonly found in cirrhotic patients [2, 9, 19].

**Table 1:** Agents forbidden in hemodynamic restitution for variceal hemorrhage

Liquid forbidden use	Reasons
Normal saline	Worsen the formation of ascites as well as other extra-vascular fluid accumulation
Dextrans	Side-effect on bleeding times
Hydroxyethyl starch	Worsen hepatic function
Ringer’s lactate solution	Contraindicated in case of liver dysfunction

Furthermore, most clinicians are opt to correct hypovolemia conservatively and cautiously [2, 3]. During the correction of hypovolemia, portal pressure increases 20% more rapidly than blood volume. Thus, over-expansion of plasma volume may result in a more severe increase in portal pressure and the risk of further bleeding subsequently increases [3]. In practice, volume placement is performed with the goal of maintaining hemoglobin at approximately 8 g/dL [9].

### Antibiotic prophylaxis

Bacterial infection is a serious complication of cirrhosis, especially in bleeding patients [2, 3]. It is reported that 30% - 40% of cirrhotic patients undergo bacterial infections during an episode of variceal hemorrhage or within the first week following bleeding [20]. Spontaneous peritonitis and bacteriaemia are the most common infections seen in cirrhotic patients. Infections alter systemic and splanchnic hemodynamics, worsen coagulation disorders, impair liver function and subsequently may induce variceal bleeding [21]. Altogether, bacterial infection leads to failure in controlling acute bleeding, early re-bleeding and death.

Prophylactic use of antibiotics has been proved to reduce the incidence of bacterial infections [2, 3, 9, 12, 20], reduce the rate of re-bleeding [9, 12], and significantly improve survival [1, 2, 8, 9, 12, 19, 20]. Therefore, antibiotic prophylaxis has been an indispensable component of the management of acute variceal hemorrhage. Norfloxacin (400 mg, twice a day) is the most conventional antibiotics [8, 9, 20]. The duration of therapy is 7 days [1, 8, 9]. It can not be used in pregnancy, lactating women and pediatric patients. The most familiar adverse reaction is hypersensitivity, especially cutaneous anaphylaxis [8]. Intravenous quinolones are applied when oral administration is not available [9]. Besides, intravenous cephalosporins, such as ceftriaxone, are used when there is advanced liver dysfunction [5]. Aminoglycosides should be avoided considering the risk of renal toxicity [9, 18].

### Hemostasis

The combination of pharmacological and endoscopic therapy is recommended as the first-line management of acute variceal bleeding [2, 9]. A meta-analysis reported that the combination reduced overall and variceal re-bleeding in cirrhosis more than either therapy alone [22]. Once there is suspicion of variceal hemorrhage, vaso-active drug should be administered as soon as possible, even preceding diagnostic endoscopy [9]. Emergency endoscopy ought to be performed within 12 h after admission and endoscopic therapy should be performed simultaneously once the suspected variceal source of bleeding is identified [5].

#### Pharmacological therapy

Pharmacological therapy plays an important role in emergency hemostasis. Vaso-active drug can temporarily decrease portal pressure and bleeding, which provides better visualization of the esophageal lumen for endoscopy. It is recommended that pharmacological therapy should last for 5 days [9]. The following we will discuss vasopressin, terlipressin, somatostatin, and octreotide.

Vasopressin (0.2 - 0.4 U/min) causes splanchnic vasoconstriction so that it reduces portal blood flow and variceal pressure [2, 19]. However, it is associated with increased risk of myocardial infarction and mesenteric ischemia. Hence, it was abandoned as a mono-therapy 25 years ago in most countries [23]. Some clinicians advocates that vasopressin in combination with nitroglycerin can be used in emergency hemostasis [2, 9]. However, the efficacy needs further studies.

Terlipressin (Triglycyl-lysl-vasopressin) is a synthetic analogue of vasopressin with a longer half-life [8, 11]. It is currently given by a 2 mg bolus every 4 h and the duration should be maintained for 2 - 5 days [20]. It is as effective as EIS in emergency treatment and secondary prophylaxis of variceal bleeding [4]. It also induces ischemic complications, including myocardia ischemia, intestinal infarction, and limb ischemia [18]. However, the complications are not as severe as vasopressin [2, 19].

Somatostatin (a 100 µg bolus followed by infusion at 50 µg/h) is a splanchnic vasoconstrictor which can significantly reduce the HVPG, variceal pressure and azygos blood flow [19]. It achieves a reduction in HVPG by 17% without affecting systemic circulation [18]. However, the half-life of natural somatostatin is about only 3 minutes which limits its application in clinical practice [23].

Octreotide (a 50 µg bolus followed by a constant infusion at 50 µg/h) is a synthetic analogue of somatostatin with a longer half-life. It is the only drug licensed for acute variceal bleeding in the United States. Severe adverse reactions are rarely seen [2, 20]. However, the efficacy of octreotide as a mono-therapy in hemostasis is still controversial [18, 20]. It seems to be more effective when combined with endoscopic therapy [8].

#### Endoscopic therapy

Emergency endoscopy is recommended as soon as possible after admission, especially in patients with clinically significant hemorrhage or in patients with features suggesting cirrhosis [3, 5, 9, 11, 12]. Emergency endoscopy can clearly identify the bleeding source which helps guide the following steps of management. In addition, endoscopic treatment could be performed simultaneously if necessary.

Endoscopic treatment is the cornerstone of management of variceal hemorrhage. Endoscopic modalities, consisting of endoscopic injection sclerotherapy (EIS) and endoscopic variceal ligation (EVL), achieve hemostasis in approximately 90% of cases [4, 24, 25]. Although the two modalities are comparable in eradication of varices, EIS is thought to be inferior in terms of re-bleeding rates, complications, numbers of sessions, and duration for eradication [1, 2, 4, 5, 12, 19, 20, 26, 27]. Thus, EVL is generally considered as the option of endoscopic therapy [5]. However, during acute bleeding, EVL is sometimes technically difficult given that the spurting blood and clot may obscure the field of view upon endoscopy. In this situation, EIS should be employed [2, 11, 18].

It is still questionable whether it is beneficial to combine the two modalities. Mohamed AR et al reported the combined therapy was inferior to either therapy alone with regarding to the re-bleeding rate [28]. A meta-analysis of endoscopic sequential ligation plus sclerotherapy (EVLS) suggests the sequential combination lead to less complications and a higher incidence of variceal eradication [29]. The totally different conclusions may be owing to different techniques of EIS, different timing of each modality, etc. In summary, the efficiency of the combined endoscopic therapy requires future studies.

EIS was first reported by Grafoord in Sweden in 1939. This technique was then widely used and had been the first-line treatment for acute variceal hemorrhage before EVL emerged. The standard strategy of EIS is still unknown despite years of research. There are wide variations in sclerosants (type, concentration, volume injected each session), intervals between sessions, site of injection (paravariceal, intravariceal, or combined), etc. Sclerosants often used include sodium morrhuate, sodium tetradecyl sulphate, ethanolamine oleate, polidocanol, absolute alcohol, thrombin, cephalothin, phenol and tissue glue [4]. Until now there is no consensus on the optimal sclerosant for EIS [2, 4]. The commonly used sclerosants are listed in table 2.

**Table 2:** Commonly used sclerosants

Sclerosant	Concentration	Volume/site (ml)	The max value of volume/session (ml)	Special points
Sodium morrhuate	5%	4-6	20	Commonly used in China
Ethanolamine oleate	5%	2-3	25	Commonly used in China
Polidocanol	1%	1-2	20	n/a
Sodium tetradecyl sulphate	0.5%-1.5%	5	n/a	Associated with more complications and seldom used now

In spite of these differences, EIS is beneficial either in emergency hemostasis or prevention of recurrent hemorrhage. However, it is associated with a series of complications, retro-sternal chest pain, ulcer, esophageal stenosis, fever, pneumonitis, dysphagia, esophygeal perforation and bacteriemia [1, 4, 8, 16, 19]. It is noteworthy that EIS can not alleviate portal hypertension. After EIS, portal hypertension still exists, which is responsible for the recurrence of varices and hemorrhage. Thus, it is necessary to perform a long term strategy of EIS until eradication of varices, and a life-long endoscopic follow-up is recommended [30].

EVL was first reported by Stiegmana in 1986. Unlike EIS, the technique of EVL is relatively identical [4]. Complications associated with EVL mainly include superficial ulcer, esophageal mucosal tears, variceal rupture, etc [1, 4, 8, 16]. Several trials have been published comparing EVL and EIS. It is reported that EVL is as effective as EIS in eradication of varices and emergent hemostasis. But EVL tends to be associated with less complications and relative lower frequency of re-bleeding [31]. A problem worthy to be pointed out is that EVL, compared to EIS, is associated with a higher incidence of recurrent varices and portal hypertensive gastropathy [32]. However, the higher incidence of recurrent varices doesn’t result in a higher risk of re-bleeding. Thus, EVL has replaced EIS in most cases and it is now the most promising choice of endoscopic therapy [27]. Repeat sessions of EVL and long-term endoscopic surveillance are also required given risk of recurrent varices and bleeding. The details will be discussed in the secondary prophylaxis of variceal bleeding.

#### Failure of the first-line therapy

Even in the best condition, the current therapy seems to be ineffective in 10% - 20% patients. When the first-line treatment fails to control acute variceal bleeding, we have to immediately change our therapeutic strategies. Thus an explicit definition is quite important. The definition of failure has undergone obviously transitions during last decades.

The Baveno I (1990) definition is not quite definite. Factors regarded include blood pressure, pulse, hematocrit and hemoglobin. But no accurate data was given. The time frame is 24 h. Bleeding that occurs after a 24-h interval from “time zero” (the time of first hospitalized) is defined as re-bleeding [33].

The Baveno II (1995) and Baveno III (2000) criteria are more detailed and painstaking [11]. The definition of failure is divided into two frames. Within 6 h: any of the following factors: (a) transfusion of 4 units of blood or more, and (b) inability to achieve an increase in systolic blood pressure of 20 mmHg or to 70 mmHg or more, and/or (c) a pulse reduction to less than 100/min or a reduction of 20/min from baseline pulse rate. After 6 h: any of the following factors: (a) the occurrence of hematemesis, (b) reduction in blood pressure of more than 20 mmHg from the 6-h point, and/or (c) increase of pulse rate of more than 20/min from the 6-h point on two consecutive readings 1 h apart, (d) transfusion of 2 units of blood or more (over and above the previous transfusion) required to increase the HCT to above 27% or Hb to above 9 g/dL.

The Baveno IV Consensus Conference (2005) offers a well developed definition of failure in emergent hemostasis [11]. The time frame for the acute bleeding episode is 120 h. That is, the very early re-bleeding (within 5 days) also implies the failure of emergency hemostais. Thus, the treatment of re-bleeding within 5 days becomes a part of emergency management. The therapy is considered to have failed in case whichever below occurs, (1) fresh hematemesis ≥ 2h after the standard therapy starts. And in patients with a naso-gastric tube in place, aspiration of greater than 100ml of fresh blood signifies failure; (2) a drop in the hemoglobulin value ≥ 3g if no transfusion is administered; (3) ABRI ≥ 0.75 at any time point (however, the threshold of defining failure requires further studies). ABRI (adjusted bleed requirement index) = (blood units transfused)/ [(final HCT-initial HCT) + 0.01]; (4) death. In case of failure, we have to turn to rescue therapies.

#### Rescue modalities

The failure of the first-line therapy generally indicates a second attempt at endoscopic treatment [1, 4, 9, 12, 18, 26, 34]. However, if the second endoscopic treatment fails, salvage modalities should be employed at once [4, 9, 11, 18, 26]. Some clinicians suggest rescue therapy be taken immediately after the initial failure [3, 11, 16, 19, 35, 36]. Rescue modalities include balloon tamponade, TIPS, PTVE and shunt surgery.

Balloon tamponade, including Minnesota tube and Sengstaken-Blakemore tube, is a temporary life-saving modality against fierce bleeding that cannot be controlled by current therapy. It can successfully achieve haemostasis in most cases [2]. However, once the balloon is deflated, bleeding recurs in 50% of the patients within 24 h [9, 26]. Thus, it usually works as a “bridge” to a more definite therapy, such as repeat endoscopic therapy, TIPS and shunt surgery [1, 4, 9]. A long-duration usage of balloon tamponade may result in esophageal ulcer or even peroration [2]. So it is mentioned that the duration should not exceed 24 h [5, 9]. Other balloon-associated complications are mainly vomiting and aspiration [9, 16]. Hence, air way protection as well as sedation is of importance when using balloon tamponade [26].

TIPS, first reported in 1988, is expensive and invasive. It is an interventional radiologic procedure. General anesthesia is not always required. A shunt is produced between the hepatic vein and the intra-hepatic portion of the portal vein. TIPS could achieve hemostasis in most cases. Thus it is now considered as the first option of rescue therapy when the combination of vaso-active drug and endoscopic treatment fails. Unlike shunt surgery, TIPS is not contraindicated for patients awaiting liver transplantation. Patients with de-compensated cirrhosis (Child’s class B or C) are recommended to receive TIPS rather than shunt surgery. Complications include portosystemic encephalopathy, stenosis of shunt, shunt thrombosis, portal venous thrombosis, bleeding, hemolytic anemia, cardiac arrhythmias, TIPS-associated biliary fistula, liver failure and renal failure [8, 16].

Over the past years, Zhang et al developed a modified percutaneous transhepatic embolization of varices (PTVE) with 2-octyl cyanoacrylate (2-OCA), in which 2-OCA was injected into the whole lower esophageal and para-esophageal varices, the submucosal varices and the adventitial plexus of the cardia and fundus, this way, not only the esophageal varices but also the feeders were obliterated sufficiently to prevent variceal recurrence and improve long-term efficacy [37, 38]. In the prospective randomized controlled trial [38], cirrhotic patients with acute or recent esophageal variceal bleeding were assigned randomly to PTVE (52 patients) or EVL (50 patients) groups. With the whole lower esophageal and peri or para-esophageal varices, the submucosal varices, and the adventitial plexus of the cardia and fundus sufficiently obliterated by 2-OCA, this modified PTVE was more effective than EVL in the management of esophageal varices recurrence and rebleeding.

Common types of shunt surgery include spleno-renal shunt, meso-caval shunt and portal-caval shunt. Shunt surgery is reserved for patients unresponsive to the first-line therapy when TIPS is not available or an attempt at TIPS has already failed. However, it seems to be a better choice than TIPS for patients with Child’ class A. Patients awaiting liver transplantation are not appropriate candidates for shunt surgery. Common complications include infection, portosystemic encephalopathy, shunt thrombosis, hehepatorenal syndrome, liver failure, multisystem organ failure [8, 16].

## IV. Secondary Prophylaxis of Variceal Hemorrhage

Endoscopic modalities can achieve haemostasis in approximately 90% of the patients. Whereas, after cession of acute esophageal variceal bleeding, the risk of rebleeding approaches 70% if further preventive measures are not taken [2, 4]. The risk of recurrent hemorrhage may be increased by several factors: fierce bleeding during the first episode of hemorrhage; presence of hepatic encephalopathy; severely increased portal pressure; large varices; presence of hepatoma, etc [2]. Hence, prevention of recurrent hemorrhage is of vital importance. All patients who have survived the first episode of bleeding should take preventive measures. In the last 3 decades, many therapies have been developed to prevent the occurrence of re-bleeding. It is proposed in recent practice guidelines that a combination of EVL plus non-selective β-blockers is the most promising strategy [5, 11]. However, pharmacological therapy alone is sufficient for patients responsive to β-blockers [17]. Regular surveillance also helps monitor the prognosis of varices.

The secondary prophylaxis starts on day 6 after the first episode [3, 11, 12, 17]. Very early re-bleeding (within 5 days of acute bleeding) has been discussed above.

### Pharmacological therapy

The efficacy of pharmacological therapy in prevention of re-bleeding has been confirmed. It is observed that patients in whom the HVPG is pharmacologically reduced to less than 12 mm Hg or a reduction in HVPG is greater than 20% from baseline are associated with a rather low re-bleeding rate [9]. Non-selective β-blockers are the most widely used agent in the secondary prophylaxis of variceal hemorrhage. It reduces the risk of recurrent hemorrhage by 40% and the mortality by 20% [26]. The mechanism has been discussed above. The addition of ISMN enhances the reduction of portal pressure but it is abandoned in monotherapy. Thus, a combination of β-blockers and ISMN is considered to be pharmacological therapy of choice. However, the combination also causes greater side effects and intolerance, which limit its application in practice [5, 17]. In summary, either the combination of β-blockers plus ISMN or β-blockers alone is beneficial. The choice of the optimal agents is virtually a “one from the two” question based on individual cases.

### Endoscopic therapy

#### EIS

EIS is confirmed to be beneficial in prevention of re-bleeding [26, 39]. Despite the inferiorities to EVL, it is still widely used all over the world. There is overwhelming consensus that EIS should be repeated until eradication. Thus, the best schedule of the long-term strategy is worth attention. Generally, initial EIS is performed at the time of diagnostic endoscopy and the second session is suggested within a week [2]. Thereafter, EIS should be repeated weekly or bi-weekly in line with current consensus [5].

Heretofore, 6 trials have been published, aiming to find the optimal schedule [40-45]. Factors taken into consideration involved duration for eradication, numbers of sessions, amount of sclerosants consumed, complications and re-bleeding rate. In 1984, Westaby D et al led a randomized control trial comparing weekly schedule and 3-week schedule. The weekly schedule was reported to be with shorter duration required for eradication but a significantly higher incidence of ulcerations [40]. Sarin’s study in 1986 showed a similar result except that the weekly schedule significantly decreased re-bleeding rate [41].

A study from Japan certified there were no significant differences between weekly and 2-week schedule in terms of most parameters except for the duration for eradication [42]. The 2-week group achieved eradication significantly earlier. Massoud’s trial offered entirely equal results [43].

There are only 2 trials focusing on intervals less than a week. Conflicting results were found. Akriviadis E proposed EIS intercalated by interval shorter than weekly was less effective and more dangerous while a study from Bombay raised the advantages of 3-day schedule with regarding to duration for eradication as well as survival [44, 45]. Utterly different techniques used in the two trials might be responsible for the collision to some extent. And limited sample capacity partially affected the outcome.

In conclusion, a weekly or bi-weekly schedule of EIS is reasonable on the basis of available evidence. After eradication, endoscopy follow-up should start, it is the same with EVL. The details of surveillance endoscopy will be discussed below.

#### EVL

EVL is the recommended form of endoscopic therapy [5]. It has been illustrated above that repeat EVL should be performed until eradication of varices. The optimal frequency of repeated EVL is still controversial. EVL is currently repeated every 7 - 14 days [5]. Gin Ho Lo raised the proposal that the schedule be modified [46]. A study from Japan in 2005 proved that EVL performed at bi-monthly intervals gained a higher total eradication rate, lower recurrence rate and lower rate of additional treatment [47]. Gavin C Harewood reported, in 2006, that a longer interval between sessions of EVL might be related to a reduced risk of re-bleeding [48]. However, evidence available now is not sufficient considering repeatability, reproducibility and sample capacities. Future studies are still required to identify the optimal schedule.

#### Endoscopy surveillance

Considering the recurrence of varices and bleeding, endoscopy surveillance is of vital importance. Screening endoscopy is recommended to be repeated every 6 - 12months after the eradication of varices [1, 2, 4, 5, 9, 49]. The follow-up should be life-long [30]. Once there is evidence of recurrent varices, a comprehensive endoscopic therapy should be initiated again [9, 12, 49]. Clinicians in China proposed a detailed schedule of endoscopy surveillance, which is illustrated in table 3.

**Table 3:** Schedule of endoscopy surverillance

Time frame	Intervals between re-endoscopy
≤ 2 years after eradication	3 - 6 months
> 2 and ≤ 3 years after eradication	6 - 12 months
> 3 years after eradication till death	12 months

Endoscopic ultrasound (EUS) is a non-invasive technique which can provide good delineation of the cross sectional anatomy around the gastro-esophageal junction, including presence of varices, size of varices, wall thickness, presence of perforating veins, etc [25, 50]. The presence of esophageal collaterals and perorating veins are thought to be correlated with the recurrence of esophageal varices in cirrhotic patients [51-55]. Thus, EUS might help predicate the recurrence of esophageal varices.

### Failure of secondary prophylaxis

**Figure 3:**
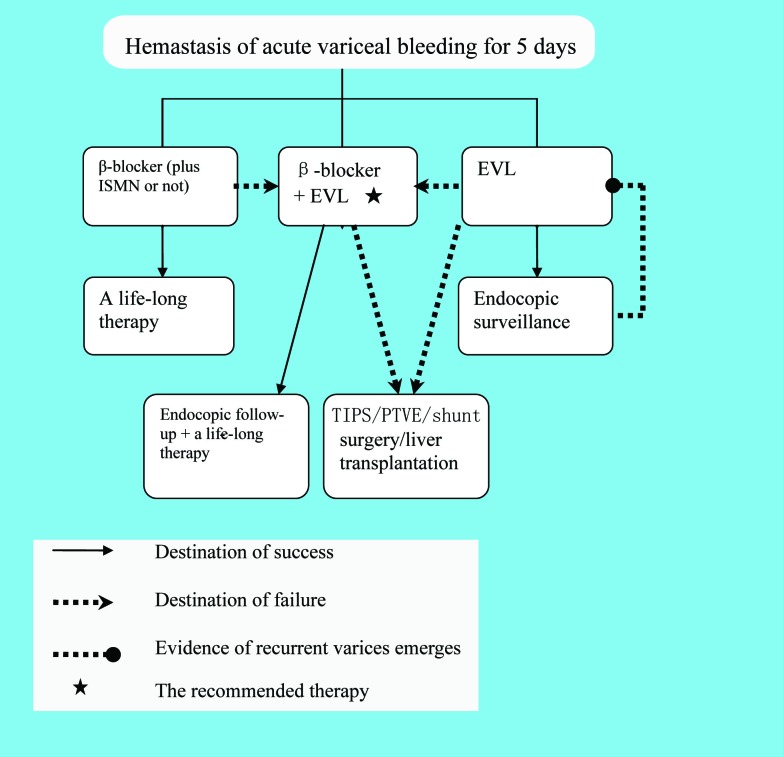
Algorithm of the secondary prophylaxis

The secondary prophylaxis is illustrated in Figure 3. The failure of secondary prophylaxis is difficult to formulate. Jake E. J. Krige et al suggested that patients who developed life-threatening variceal hemorrhage after an adequate course of treatment should be regarded as failures of long-term therapy [49]. However, re-bleeding also requires alternative treatment options in case that a patient is receiving the combined therapy (EVL + β-blocker) [9]. Salvage modalities (TIPS, PTVE or shunt surgery) should be used in case of failure [4, 9, 11, 26, 49]. TIPS is usually the preferred therapy [9]. If necessary, liver transplantation could be taken into consideration as well [9]. It is worth notice that portacaval shunts should be avoided in candidates for transplantation [2].

## V. Conclusions

Variceal hemorrhage is a life-threatening complication of portal hypertension. The management can be divided into 3 parts, primary prophylaxis, emergency treatment, and secondary prophylaxis. Screening endoscopy is recommended once the diagnosis of cirrhosis is established. Non-selective β-blocker, plus ISMN or not, is the first-line choice for patients with large varices. EVL is the alternative option when β-blocker is contraindicated or in-tolerated. Once there is a suspicion of variceal bleeding, hemodynamic restitution should be initiated as soon as possible. Volume replacement should be cautiously performed since overload of volume may lead to a severe increase in portal pressure and subsequently the risk of further bleeding. A 7-day course of prophylactic antibiotics decreases the incidence of re-bleeding and significantly improves survival. Norfloxacin, 400 mg twice a day, is the current option. Emergency endoscopy is recommended within 12 h after admission and endoscopic therapy should be performed once the suspected variceal source of bleeding is identified. The combination of vaso-active drug and EVL is recommended as the first-line management of acute variceal bleeding. EIS can be administered when EVL is technically infeasible. The failure of current therapy requires a second attempt at endoscopy therapy or rescue therapy. Balloon tamponade works as a bridge to more definitive measures (e.g, TIPS PTVE or shunt surgery); TIPS or PTVE is the option of salvage measures. Combination of non-selective β-blockers plus EVL is the best option of secondary prophylaxis. After the eradication of varices, a life-long follow-up upon endoscopy is administered. Moreover, newly developed techniques, such as ECE and EUS, may play an important part in the future and requires further studies.
